# Chemical and Radiological Characterization of Serbian Peloids: Implications for Therapeutic Safety

**DOI:** 10.3390/toxics14050355

**Published:** 2026-04-23

**Authors:** Tijana Mutić, Tijana Milićević, Emilija Vukićević, Jovana Roganović, Gorica Veselinović, Marija Janković, Gordana Gajica

**Affiliations:** 1 Institute of Chemistry, Technology and Metallurgy—National Institute of the Republic of Serbia, University of Belgrade, Njegoševa 12, 11000 Belgrade, Serbia; gorica.veselinovic@ihtm.bg.ac.rs (G.V.); gordana.gajica@ihtm.bg.ac.rs (G.G.); 2 Institute of Physics Belgrade—National Institute of the Republic of Serbia, University of Belgrade, Pregrevica 118, 11080 Belgrade, Serbia; tijana.milicevic@ipb.ac.rs; 3 Faculty of Chemistry, University of Belgrade, Studentski trg 12-16, 11158 Belgrade, Serbia; emilija@chem.bg.ac.rs (E.V.); jovanaorlic@chem.bg.ac.rs (J.R.); 4 VINČA Institute of Nuclear Sciences—National Institute of the Republic of Serbia, University of Belgrade, Mike Petrovića Alasa 12-14, 11000 Belgrade, Serbia; marijam@vin.bg.ac.rs

**Keywords:** therapeutic muds, balneotherapy, elemental composition, natural radionuclides, risk assessments

## Abstract

Peloids are natural materials widely used in balneotherapy and dermatological treatments because of their physicochemical and mineralogical properties. Despite Serbia’s long tradition of spa-based pelotherapy, comprehensive data on the chemical and radiological characteristics of local peloids remain limited. In this study, peloid samples from 13 spa locations across four regions of Serbia were systematically investigated. The aim was to determine their physicochemical properties, elemental composition, and natural radioactivity, to assess their suitability and safety for therapeutic use. The analyzed samples exhibited pronounced variability in pH (6.59–9.52), electrical conductivity (77.5–6610 μS/cm), salinity (below detection limit to 4%), and total dissolved solids, reflecting diverse geological and hydrochemical properties. Inductively coupled plasma optical emission spectrometry revealed site-specific variations in macro- and microelements, influenced primarily by local lithology and sedimentary environments, with limited indications of anthropogenic inputs. Gamma spectrometric analysis showed that the activity concentrations of naturally occurring radionuclides (^226^Ra, ^232^Th, ^40^K, ^238^U, ^235^U, ^210^Pb) were within ranges commonly reported for therapeutic muds worldwide, while anthropogenic ^137^Cs was generally low. Radiological hazard indices were below internationally recommended safety limits. A preliminary screening of dermal exposure to potentially toxic elements indicated no significant noncarcinogenic risk (HI < 1) and acceptable carcinogenic risk (TCR) levels. Overall, this study provides a comprehensive chemical and radiological baseline for Serbian peloids, supporting their safe use in controlled therapeutic and wellness applications and highlighting the importance of site-specific characterization for quality assessment.

## 1. Introduction

Peloids have been used since ancient times for their therapeutic properties, for ritual practices, and for physical rehabilitation, with historical records dating back to the civilizations of Egypt, Greece, and Rome [1,2]. Peloids, commonly known as therapeutic or healing muds, are sticky, pliable natural materials formed through complex interactions between mineral water, inorganic sediments, and organic matter of plant or microbial origin [3]. They are composed of a liquid phase represented by mineral-rich water, and a solid phase, consisting of both organic and inorganic components. The beneficial effects of peloids in various treatments are attributed to their physicochemical characteristics, including mineralogical composition, grain size, cation-exchange capacity, pH, and levels of bioactive organic compounds [4]. These properties play a crucial role in their effectiveness for thermotherapy, as well as their neuromuscular, analgesic, immunological, and chondroprotective effects [5,6,7]. Beyond therapeutic use, peloids are also applied in dermatology and cosmetology. Their fine-grained texture and bioactive composition support skin hydration, exfoliation, rejuvenation, and the management of inflammatory or microbial skin conditions [8,9,10]. This wide-ranging applicability highlights the interdisciplinary relevance of peloid research, spanning geosciences, chemistry, medicine and wellness sciences.

The therapeutic potential and safety of peloids can vary significantly between regions, depending on local geology, hydrochemistry, and organic contributions, such as plant or microbial input [4]. Contaminants such as heavy metals or pathogenic microorganisms may be present, posing health risks if uncharacterized peloids are applied clinically [11]. Globally, extensive research has been carried out to characterize peloids for therapeutic use. In Brazil, the physicochemical, mineralogical, elemental, and radiological properties of Peruibe Black Mud have been studied [10], while Torrecilha et al. compared peloids from different regions in Brazil, identifying candidates suitable for therapeutic application [12]. In Romania, the peloids of Techirghiol Lake have been extensively characterized for their chemical and radiometric properties [8]. Similarly, Roca Jalil et al. examined natural and artificially matured peloids from the Copahue thermal system in Argentina [13]. Spanish researchers have investigated peloids from various clay deposits, emphasizing their mineralogical and chemical variability and therapeutic potential [14,15]. Studies have also been conducted on peloids from Mongolia’s Lake Khyargas [16], the Hupo Basin in South Korea [9], and several regions in Turkey [17]. Collectively, these studies illustrate a global effort to characterize peloids, establish quality parameters, and expand medical and cosmetic peloid applications.

In contrast, systematic investigations of peloids in the Balkans remain limited, despite the region’s rich geological diversity and long-standing spa traditions. In Serbia, numerous thermal springs and mineral-rich mud deposits have been historically associated with healing practices. While these resources are frequently utilized in spa treatments and traditional medicine, their mineralogical, chemical, and radiological properties are still insufficiently characterized in the scientific literature. The lack of systematic evaluation raises uncertainties regarding their reproducibility, efficacy, and potential risks, particularly in the presence of toxic elements or radioactive elements. Furthermore, without standardized quality criteria, the integration of Serbian peloids into evidence-based medicine and international therapeutic practices remains limited.

This study addresses this knowledge gap by investigating the elemental composition of peloids collected from 13 spas across four regions of Serbia. The main aim was to comprehensively characterize their physicochemical properties, macro- and microelement concentrations, and radionuclide levels, and to evaluate potential environmental and health risks associated with their use. By providing a systematic dataset, this study supports the safe and evidence-based application of Serbian peloids in clinical, wellness, and cosmetic contexts.

## 2. Materials and Methods

### 2.1. Study Area

**Figure 1:**
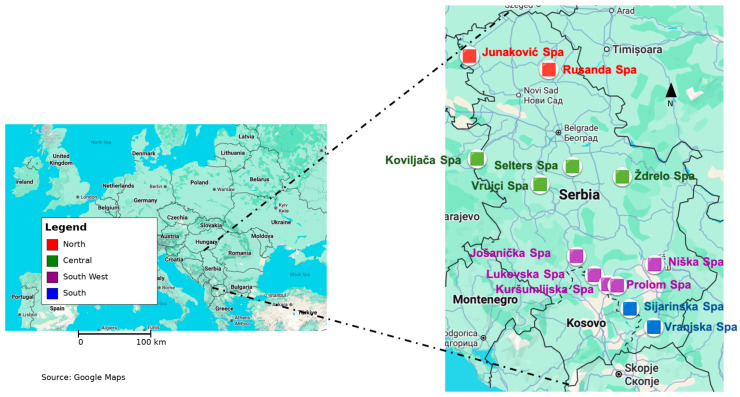
Sampling sites of peloids across four regions in Serbia: north—Junaković (JUN) and Rusanda (RUS) spas; central—Koviljača (KOV), Ždrelo (ZDR), Selters (SEL) and Vrujci (VRU) spas; ssouth-west—Niška (NIS), Prolom (PRO), Kuršumliljska (KRS) and Lukovska (LUK) spas; and South—Sijarinska (SIJ) and Vranjska (VRA) spas.

The study area includes selected spa locations across the Republic of Serbia where natural peloids are traditionally used for pelotherapy and medical treatments. Peloid samples were collected from 13 spas distributed across four geographical regions of Serbia: the northern, central, southwestern, and southern parts of the country (Figure 1). A full description of the sampling sites and location properties is given in Section 1 of the Supplementary Information (S1. Study area).

### 2.2. Samples

Sampling was conducted directly at the spas to ensure that the collected materials were representative of those applied in routine therapeutic practice. Depending on the local spa protocol, three types of peloid materials were sampled. In seven spas—Junaković (JUN); Koviljača (KOV); Kuršumlijska (KRS); Lukovska (LUK); Prolom (PRO); Niška (NIS); and Vranjska (VRA)—peloids were prepared by mixing locally sourced geological material, primarily clay- and zeolite-rich soils, with mineral water from the respective spa. These mixtures were sampled after homogenization, and used for therapeutic application. In four spas—Ždrelo (ZDR); Selters (SEL); Jošanička (JOS); and Sijarinska (SIJ)—samples were collected directly from established mud pools, where geological material and mineral water undergo natural maturation for prolonged periods, allowing extensive interaction between water and sediment. Two additional samples were obtained from natural aquatic environments used in pelotherapy. The Vrujci Spa sample (VRU) was collected from the spa river, while the Rusanda Spa sample (RUS) was collected from Lake Rusanda, a saline lake traditionally used for peloid-based treatments. All samples were collected using clean, inert polyethylene containers. The collected peloid samples (1–2 kg) were air-dried at room temperature for three weeks, homogenized, sieved (Ø 2 mm), and finely ground to obtain a homogeneous powder.

### 2.3. Physicochemical Analysis of Peloids

Physicochemical parameters, including pH, electrical conductivity (EC), total dissolved solids (TDS), and salinity, were determined following standardized procedures commonly applied for soil and sediment materials [18]. Measurements were performed on aqueous suspensions prepared by mixing 10 g of dried peloid with 50 mL of Milli-Q water, followed by continuous stirring for 1 h at room temperature to allow equilibration. pH measurement of peloid samples was performed using a pH 50 VioLab Benchtop pH Meter with a 201T pH electrode (XS Instruments, Giorgio Bormac, Carpi, Italy). EC, TDS, and salinity measurements were done on a Handheld meter Cond 330i/340i (WTW, Weilheim, Germany) at room temperature.

### 2.4. Elemental Concentrations of Peloids

The elemental concentrations were determined using inductively coupled plasma optical emission spectrometry Thermo Fisher iCAP 6500 (Thermo Fisher Scientific, Waltham, MA, USA). All chemicals used were of analytical grade, purchased from Sigma Aldrich (St. Louis, MO, USA), and used without purification. For ICP-OES analysis, nitric acid (70%, ACS reagent) and hydrochloric acid (37%, ACS reagent) were used. The certified reference material ICP Multi-element standard solution IV (1000 mg/L of 23 elements: Ag, Al, B, Ba, Bi, Ca, Cd, Co, Cr, Cu, Fe, Ga, In, K, Li, Mg, Mn, Na, Ni, Pb, Sr, Tl, and Zn) was used for standard solution preparation in a concentration from 1 to 1000 μg/L for microelements and 1 to 25 mg/L for macroelements. The blank used in these measurements was an aqueous solution of 2% (*v*/*v*) nitric acid prepared from concentrated nitric acid. For ICP-OES analysis, the samples underwent wet digestion. A total of 0.5 g (accuracy ± 0.1 mg) of each sample was treated with 20 mL of aqua regia (a 1:3 *v*/*v* of nitric acid and hydrochloric acid) and heated at 80 °C for 8 h. Once the digestion was nearly complete and approximately 2 mL of solution remained, the samples were filtered and diluted to 50 mL with distilled water. The samples were analyzed in triplicate under the same conditions as the standards (adjusted acid concentration). In all measurements, Milli-Q distilled water was used.

### 2.5. Radionuclide Concentrations of Peloids

For gamma spectrometric measurements, samples were dried in an oven at a temperature of 105 °C to a constant mass. Analysis was performed using a High-Purity Germanium (HPGe) detector with a relative efficiency of 18% (Gama spectrometer 7229N-7500-1818, Canberra Industries, Inc. (Mirion Technologies, Meriden, CT, USA)). The calibration of the detector was performed using a certified radioactive standard in Marinelli geometry (1035 SE-40845-17, Czech Metrology Institute, Inspectorate for Ionizing Radiation, Prague, Czech Republic) which is traceable to BIPM. The measurement duration was 60,000 s. The spectra were analyzed using GENIE 2000 software. The measurement results are expressed as Bq/kg with a confidence level of 95% (k = 2). The specific activities of the radionuclides were detected using gamma energies of 295 keV, 352 keV, 609 keV, 1120 keV, and 1764 keV (the activity of ^226^Ra); 338 keV and 911 keV (the activity of ^232^Th); 1460 keV (^40^K); 143 keV and 186 keV (^235^U), which were corrected for the contribution from ^226^Ra, 63 keV; 1000 keV (^238^U); and 46 keV (^210^Pb). The activity of artificial radionuclide ^137^Cs was determined using an energy of 661 keV. Background spectra were recorded under identical measurement conditions using an empty Marinelli container and subtracted from the sample spectra before activity calculation.

The quality of the measured results, in terms of accuracy and precision, depends on properly performed energy and efficiency calibration. All radionuclide analyses were carried out in an accredited laboratory in accordance with ISO/IEC 17025 [19], ensuring the reliability and traceability of the results. External quality assurance was verified through regular participation in proficiency testing (PT) schemes. The Radiation and Environmental Protection Department, Vinča Institute of Nuclear Sciences, National Institute of the Republic of Serbia, University of Belgrade, has extensive experience in PT programs organized by the IAEA, with consistently satisfactory performance. The reported results showed good agreement with the reference values, confirming the accuracy and precision of the applied methodology [20].

### 2.6. Data Analysis and Health Risk Assessment

The normality of the data was tested by the Kolmogorov–Smirnov test, and Spearman’s correlation analysis was done using Origin Pro 2025 with a Python plug-in. The element and radionuclide distribution were evaluated by factor analysis.

The dermal noncarcinogenic and carcinogenic risks were assessed for adults who use peloids as therapy for a long time. The equations used for human health risk assessment are equations developed by the Oak Ridge Operations Office [21] U.S. Department of Energy (DOE), and were adopted and adapted for dermal exposure to polycyclic aromatic hydrocarbons (PAHs). Estimated daily intake for noncarcinogenic (EDI_nc_) and carcinogenic (EDI_c_) substances were determined using formulas explained previously, and their detailed adaptation will be discussed in further studies. The total hazard index (THQ) and carcinogenic risk reported as the total cumulative cancer risk (TCR) were assessed in the following way [22,23]:

$$\mathrm{THQ}\;=\;\frac{EDInc}{RfD}$$

HI = ∑THQ

TCR = EDI_c_ × CSF

TCR = ∑CR

where EDI_nc_ and EDI_c_ are the estimated daily dermal intake of elements that were assessed by using the equations used for human health risk assessment developed by the Oak Ridge Operations Office, U.S. Department of Energy (DOE); RfD is the reference dose value; and CSF is the cancer slope factor. The exposure frequency for the equation is 20 days × year^−1^, the exposure duration is 26 years, the exposed skin area is 6.032 cm^2^ × day^−1^, the peloid adherence factor is 0.12 mg × cm^−2^, and a body weight of 80 kg was used for adults.

In parallel, a few radiological indices were calculated. The radium equivalent activity was calculated to compare the specific activities of materials using the following equation [24]:

Ra_eq_ = C_Ra_ + 1.43C_Th_ + 0.077C_K_

C_Ra_, C_Th,_ and C_K_ represent the activity concentrations for the three radionuclides in Bq/kg. Another important parameter is the absorbed dose rate (D_out_), which takes into consideration the contribution of gamma radiation from naturally occurring radionuclides ^226^Ra, ^232^Th, and ^40^K, calculated using the following equation [25]:

D_out_ = 0.462C_Ra_ + 0.604C_Th_ + 0.0417C_K_

The conversion factors for ^226^Ra, ^232^Th, and ^40^K are 0.462, 0.604, and 0.0417 nGyBq/hkg, respectively, with an assumption that other radionuclides have a negligible contribution [8]. The annual effective dose (AED) was calculated using the following equation [26]:

AED (μSv/y) = D_out_ (nGy/h) × 0.2 × 24 (h) × 365.25 (d) × 0.7 μSv/y × 10^−3^

where 0.2 represents 20% outdoor occupancy, 0.7 μSv/y is a conversion factor between the absorbed dose ratio and annual effective dose, and 24 (h) and 365.25 (d) represent hours and days. The gamma radiation hazard index (I_ϒ_r), a significant health index that estimates the excess external and indoor gamma radiation, was calculated using the following equation [27]:

I_ϒ_r = C_Ra_/150 + C_Th_/100 + C_K_/1500

The external hazard index (H_ex_) and internal hazard index (H_in_) were calculated using the following equations [28]:

H_ex_ = C_Ra_/370 + C_Th_/259 + C_K_/4810

H_in_ = C_Ra_/185 + C_Th_/259 + C_K_/4810

The excess lifetime cancer risk (ELCR) was evaluated using the following equation [26]:

ELCR = AED (μSv/y) × DL (y) × RF (1/Sv)

where DL represents a lifetime of 70 years, and RF describes the fatal cancer risk factor of 1/Sv. These parameters represent standardized probabilistic risk assessment indicators widely applied in environmental health and radiological protection studies. They do not represent clinical diagnoses but provide a statistical estimation of potential health risk under defined exposure scenarios.

## 3. Results

### 3.1. Physicochemical Analysis

The analyzed peloids exhibited pronounced variability in physicochemical properties, reflecting the diverse geological and hydrochemical environments from which they originate. The results of the physicochemical analysis are presented in Table 1.

The observed pH values ranged from slightly acidic (6.59) at the Vrujci Spa to strongly alkaline (9.52) at the Rusanda Spa, which is consistent with previously reported data for therapeutic muds worldwide and can be attributed primarily to mineralogical composition and the chemistry of associated mineral waters. Alkaline conditions are commonly associated with carbonate-rich lithologies and evaporitic environments [29]. Some studies suggest that mild alkaline muds may contribute to beneficial skin responses during controlled therapeutic treatments, including effects on skin hydration and epidermal functions [30]. However, because the skin surface is slightly acidic, the interaction between mud pH and the protective acid mantle of the skin should be carefully considered when evaluating their dermatological applications [31].

The EC values of peloids varied widely from 77.5 μS/cm at the Ždrelo Spa to 6610 μS/cm at the Rusanda Spa. Elevated EC values reflect higher ionic strength, which enhances the mineral exchange processes between peloids and the skin during therapy. The Rusanda Spa, with its exceptionally high EC, stands out as an outlier, consistent with its saline lake origin [32]. Salinity was under the detection limit (<0.1%) in most samples, except for the Rusanda Spa (4%), Junaković Spa (0.4%), and Selters Spa (0.4%). Elevated salinity levels, particularly at the Rusanda Spa, can be attributed to intense evaporation and halite precipitation. Although high salinity can stimulate circulation and promote detoxification via osmotic gradients, excessive concentrations may pose risks of irritation or dehydration, signifying the importance of balancing therapeutic gain with safety [33]. TDS values ranged from 43 mg/L at the Ždrelo Spa to 742 mg/L at the Junaković Spa. The Rusanda Spa presented an extreme value (>1999 mg/L, above instrumental limits), consistent with its high salinity and evaporitic character. Elevated TDS values are typically associated with enhanced therapeutic properties, resulting from increased mineral availability. However, excessively high concentrations may limit tolerability, especially for individuals with sensitive skin types [34].

Overall, the physicochemical characterization highlights the heterogeneity of Serbian peloids, ranging from dilute, low-mineral muds (Ždrelo Spa and Vrujci Spa) to highly mineralized, saline muds (Rusanda Spa). This diversity reflects contrasting depositional environments and hydrochemical regimes, which in turn influence their therapeutic potential and suitability for different clinical applications.

### 3.2. Elemental Composition of Peloids

The concentrations of eight macroelements (Al, Ca, Fe, K, Mg, Na, P, and S) are presented in Table 2. Overall, the elemental concentrations of Serbian peloids show similarities with therapeutic muds reported from other well-known spa environments, while also reflecting distinct local geochemical controls. In comparison with peloids from the Dead Sea (Jordan), our data show broadly similar concentrations of Al (44.3 ± 1.19 mg/g), P (1.3 ± 0.01 mg/g), and S (1.4 ± 0.03 mg/g) [35]. However, K concentrations in Dead Sea peloids (22.2 ± 0.20 mg/g) were notably higher than in most Serbian samples (Table 2), suggesting that the K-rich evaporitic setting of the Dead Sea provides a distinctive geochemical fingerprint. Techirghiol Lake peloids (Romania) also displayed elevated K levels (12.1 ± 0.3 mg/g) [8], comparable to Serbian samples from the Rusanda Spa (32.32 ± 0.46 mg/g), where saline lake conditions clearly promoted alkali enrichment. Ca concentrations in Serbian peloids (Table 2) were substantially lower than those reported for the Dead Sea (180.0 ± 1.67 mg/g) and Techirghiol Lake (94.7 ± 7.5 mg/g). The highest Ca content in our dataset was observed at the Junaković Spa with a value of 100.66 ± 0.50 mg/g, which aligns with carbonate-rich sedimentary environments and local limestone input. Conversely, Ždrelo Spa displayed the lowest Ca concentration (6.96 ± 0.05 mg/g), reflecting its diluted mineral input and lower carbonate contribution.

Serbian peloids also exhibit significant variability in their macroelement composition. For instance, Fe concentrations varied significantly, from as low as 11.11 ± 0.08 mg/g at the Niška Spa to 124.55 ± 0.39 mg/g at the Jošanička Spa. The enrichment at the Jošanička Spa is consistent with the presence of Fe-bearing minerals such as hematite and goethite in the catchment area. Elevated Fe is therapeutically relevant, as iron oxides enhance peloid heat retention capacity, a desirable property for thermotherapy [14]. Al concentrations also spanned a wide range, from only 8.95 ± 0.25 mg/g at the Junaković Spa to 78.19 ± 0.39 mg/g at the Vranjska Spa. Such variations likely reflect contrasting contributions from aluminosilicate clays, which are dominant in some basins but diluted in carbonate-rich systems. Since Al-rich clays control cation-exchange capacity, these differences may directly influence peloid reactivity and ion-exchange potential during therapy [15]. Na and K concentrations reached their highest levels at the Rusanda Spa (30.35 ± 0.24 mg/g and 32.32 ± 0.46 mg/g, respectively), consistent with its saline lake environment and evaporitic mineral assemblage. Such enrichment in alkaline elements enhances osmotic properties, which can stimulate microcirculation during pelotherapy. Elevated P and S contents were also observed at the Rusanda Spa (3.04 ± 0.01 mg/g and 8.72 ± 0.03 mg/g, respectively), far exceeding the values in other Serbian peloids. Sulfur is particularly important for dermatological applications, as sulfur compounds are known for their keratolytic, antibacterial, and anti-inflammatory effects [8].

The microelement profiles of Serbian peloids exhibited distinct patterns across the studied spas, reflecting both geological background and local environmental influences. The results of the ICP-OES analysis of fifteen microelements are presented in Table 2. Peloids from Lixouri, Greece, exhibited results that are in agreement with ours for As (9.3–14.1 mg/kg), Ba (222–315 mg/kg), Co (8.7–20.3 mg/kg), Cu (9–35 mg/kg), and Zn (62–84 mg/kg) [36]. On the other hand, concentrations of Cr, Ni, and Sr were higher than ours (98–220 mg/kg, 55–145 mg/kg, 195–369 mg/kg, respectively), while Pb was slightly lower (11–15 mg/kg).

To distinguish between geogenic and anthropogenic sources of potentially toxic elements (PTEs), the obtained concentrations were compared with regional background values for soils in central Serbia. According to Mrvić et al. (2019), the approximate background limits (Median + 2MAD) are as follows: As (14.7 mg/kg), Cr (72.3 mg/kg), Cu (38.4 mg/kg), Ni (84.7 mg/kg), Pb (69.3 mg/kg), and Zn (68.1 mg/kg) [37]. In addition, the typical ranges of natural variation (90–95 percentiles) are as follows: As (16–25 mg/kg), Cr (81–151 mg/kg), Cu (46–65 mg/kg), Ni (95–167 mg/kg), Pb (71–88 mg/kg), and Zn (79–96 mg/kg) [37].

Concentrations of As ranged from 1.23 ± 0.90 mg/kg at the Sijarinska Spa to 45.26 ± 0.64 mg/kg at the Jošanička Spa. In several locations, particularly the Jošanička Spa, As concentrations significantly exceeded regional background levels, suggesting a strong geogenic contribution associated with hydrothermal fluids and As-bearing minerals. Ba concentrations were generally high, especially at the Niška Spa (717.46 ± 7.49 mg/kg) and Vranjska Spa (763.7 ± 3.5 mg/kg), which can be attributed to natural enrichment from Ba-rich carbonates and evaporitic deposits. Cd and Pb, which are commonly associated with anthropogenic contamination, were elevated at several spas. The highest Cd concentrations were observed at the Ždrelo Spa (3.52 ± 0.01 mg/kg) and Junaković Spa (1.55 ± 0.03 mg/kg), exceeding typical background levels by an order of magnitude, indicating a likely anthropogenic contribution, possibly from agricultural practices or local industrial activities. Similarly, Pb was notably enriched at the Ždrelo Spa (59.73 ± 0.30 mg/kg) and Selters Spa (49.11 ± 0.22 mg/kg), surpassing background values and suggesting additional anthropogenic inputs. Concentrations of Hg were generally low (<0.29 mg/kg) in the investigated peloids, except at the Sijarinska Spa (2.35 ± 0.62 mg/kg), where values exceeded typical background levels. This enrichment may reflect either a geogenic source related to regional hydrothermal and volcanic activity or local anthropogenic inputs. Considering that peloids are applied dermally and that mercury in hydrothermal environments is often associated with poorly soluble sulfide phases, its bioavailability is expected to be limited [38].

Transition metals such as Co, Cu, Mn, and Ni also displayed site-specific variability. Mn, Cr, and Ni concentrations were highest at the Selters Spa (1152 ± 12, 132.7 ± 9.49, and 148.3 ± 0.61 mg/kg, respectively), reflecting underlying mafic or ultramafic lithologies in the catchment area. Sr was significantly enriched at the Rusanda Spa (98.3 ± 2.4 mg/kg) and Sijarinska Spa (289 ± 1.7 mg/kg), in line with the elevated alkaline earth metals typically observed in saline lake environments. Li concentrations were generally low but showed moderate enrichment at the Rusanda Spa (27.3 ± 0.16 mg/kg), consistent with its evaporitic origin.

Overall, the distribution of PTEs in Serbian peloids reflects a combination of natural geochemical background and localized anthropogenic influences. Elements such as As, Ba, Sr, and partially Hg are primarily controlled by geological and hydrothermal processes, whereas Cd and Pb show clear evidence of anthropogenic contribution in certain spa locations.

### 3.3. Radiometric Results

Radionuclide concentrations of seven radionuclides in peloid samples were determined and are presented in Table 3. Natural radionuclides and anthropogenic radionuclide ^137^Cs were identified and the results revealed notable variability across different spas, reflecting local geology, sediment composition, and historical environmental inputs. The average activity concentrations of natural and anthropogenic radionuclides ^40^K, ^235^U, ^238^U, ^232^Th, ^226^Ra, ^137^Cs, and ^210^Pb were 590 ± 40 Bq/kg, 2.6 ± 0.3 Bq/kg, 41 ± 6 Bq/kg, 53 ± 4 Bq/kg, 40 ± 2 Bq/kg, 14.3 ± 1.1 Bq/kg, and 47 ± 6 Bq/kg, respectively. The radionuclide activity concentrations confirm that the studied peloids are within safe radiological limits for therapeutic use [39]. The levels of ^226^Ra (26–68 Bq/kg), ^232^Th (36–75 Bq/kg), and ^40^K (410–1060 Bq/kg) are comparable to the worldwide averages for soils (^226^Ra = 35 Bq/kg, ^232^Th = 30 Bq/kg, ^40^K = 400 Bq/kg; Ref. [39]).

Radionuclide concentrations in Turkish peloids are higher than the concentrations obtained in peloids examined in this work, with values of 110.66 Bq/kg for ^226^Ra, 445.36 Bq/kg for ^232^Th, and 1698.60 Bq/kg for ^40^K [40]. A lower activity concentration of ^137^Cs was observed in Turkish peloids, with an average value of 0.447 Bq/kg. ^40^K was the most abundant naturally occurring radionuclide, with values ranging from 410 ± 30 Bq/kg at the Prolom Spa to 1060 ± 70 Bq/kg at the Rusanda Spa. A slightly elevated ^40^K in Rusanda and Koviljača (720 ± 50 Bq/kg) is typically associated with K-rich clay minerals and feldspars, which are common components of peloids and contribute to their heat retention and thermal conductivity during pelotherapy [14]. ^226^Ra concentrations ranged from 26 ± 1 Bq/kg at the Niška Spa to 68 ± 4 Bq/kg at both the Koviljača and Selters Spas. ^232^Th activity showed a similar pattern, varying from 36 ± 3 Bq/kg at the Jošanička Spa to 75 ± 5 Bq/kg at the Vranjska Spa. Uranium isotopes (^238^U and ^235^U) were present in low concentrations, with ^238^U ranging from 23 ± 4 Bq/kg at the Vranjska Spa to 76 ± 7 Bq/kg at the Selters Spa, and ^235^U activities were consistently low (1.0–5.8 Bq/kg), reflecting the natural isotopic ratio of uranium. These variations are primarily linked to local lithological differences, as areas rich in granitic and sedimentary rocks often contain elevated Ra and Th levels [16,17]. ^210^Pb concentrations were generally moderate, ranging from 22 ± 4 Bq/kg at the Sijarinska and Vranjska Spas to 160 ± 10 Bq/kg at the Rusanda Spa. Higher concentrations may reflect the accumulation of atmospheric deposition over time in the surface mud layers, which is common in semi-enclosed thermal basins. The anthropogenic radionuclide ^137^Cs was below detection limits in most samples, or with very low concentrations ranging from 1.4 ± 0.3 Bq/kg in Ždrelo Spa to 7.0 ± 0.6 Bq/kg in Jošanička Spa, with an exception at the Rusanda Spa, where the concentration was 47 ± 3 Bq/kg. This is consistent with residual fallout from historical nuclear activities and global atmospheric deposition, suggesting minimal radiological impact on therapeutic use. Overall, the radiological profile of Serbian peloids indicates that naturally occurring radionuclides are within ranges typically reported for therapeutic muds worldwide, and ^137^Cs levels are negligible, confirming the safety of these peloids for clinical and wellness applications. The variability in radionuclide activity emphasizes the importance of site-specific characterization for both health and regulatory considerations [9].

In order to further interpret the observed variations in radionuclide activity concentrations and better understand their geochemical behavior, activity ratios among members of the ^238^U and ^232^Th decay series were evaluated. In natural environmental matrices, activity ratios among radionuclides of the ^238^U and ^232^Th decay series are generally stable and reflect their common geochemical origin [41]. Under conditions of secular equilibrium, the activity ratio between a parent nuclide and its progeny within the ^238^U series (e.g., ^238^U/^226^Ra) approaches unity, providing a benchmark for assessing geochemical consistency. Deviations from unity indicate disequilibrium, which may result from differential mobility, selective leaching, or other environmental processes affecting radionuclide distribution [42,43]. Similarly, activity ratios between members of the ^232^Th and ^238^U series, such as ^232^Th/^226^Ra, typically remain near unity in undisturbed natural systems. Variations in these ratios can reveal insights into radionuclide migration, soil–water interactions, and the geochemical behavior of parent and daughter nuclides. For instance, uranium isotopes tend to be more mobile in oxidizing environments, whereas radium is generally less mobile and strongly adsorbed onto mineral surfaces, which may lead to local disequilibrium in some environmental compartments.

Table S1 presents the ^238^U/^226^Ra and ^232^Th/^226^Ra activity ratios. The mean value for the ^238^U/^226^Ra activity ratio is 1.11. Due to the higher activity concentrations of ^238^U than ^226^Ra in most samples, the ratio is above 1, and these two radionuclides are in disequilibrium due to the different mobility of these radioisotopes. The calculated ^232^Th/^226^Ra activity ratio has a mean value of 1.41. As it is reasonable to assume that the two-decay series are in secular equilibrium in the rocks, this indicates that the ^226^Ra-to-^232^Th relationships are not directly inherited from the parent material but probably result from secondary processes (weathering).

The ^235^U/^238^U activity ratio serves as a robust indicator of uranium origin in environmental studies. Ratios consistent with natural isotopic composition confirm a geogenic source, while significant departures from this ratio can indicate anthropogenic contributions, such as depleted or enriched uranium. Overall, the combined evaluation of these activity ratios provides a powerful tool for tracing uranium- and thorium-series radionuclides in environmental studies and understanding their geochemical behavior. The natural activity concentration ratio of ^235^U/^238^U is 0.046. A lower value can indicate the presence of depleted uranium in the environment [44]. The evaluation of ^235^U/^238^U ratios, when combined with other decay-series activity ratios such as ^238^U/^226^Ra and ^232^Th/^226^Ra, provides a comprehensive framework for assessing radioactive equilibrium and geochemical behavior of uranium and thorium in the environment. Lower-than-natural ^235^U/^238^U ratios have been widely used in recent studies as a reliable marker for depleted uranium contamination in soil, water, and biota, as documented in Serbia and other regions affected by military or industrial uranium use [44].

### 3.4. Association and Origin Assessment of PTE, Physicochemical Parameters and Radionuclides

**Figure 2:**
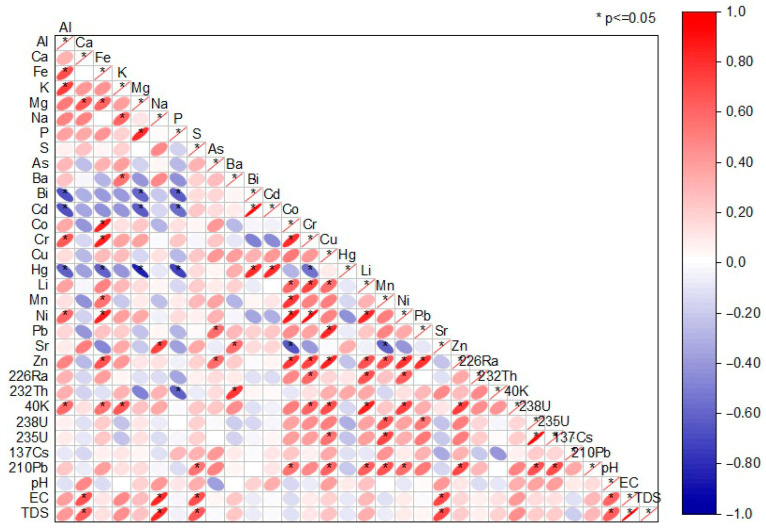
Correlogram indicating the significant correlations between element and radionuclide concentrations and physicochemical parameters of peloids in Serbia.

The correlogram (Figure 2) illustrates the significant correlations among major and trace elements, radionuclides, and physicochemical parameters in the studied peloids, reflecting coupled lithological, geochemical, and post-depositional processes.

Strong positive correlations among major elements (Ca, K, Mg, Na, Fe, Al, P, and S; Figure 2) indicate a dominant lithogenic origin and association with aluminosilicate and carbonate minerals. The Ca-Mg correlation supports the presence of carbonate minerals such as calcite and dolomite, while K, Na, Al and Fe associations indicate contributions from feldspars, micas, clay minerals, and Fe-bearing silicates, typical of mixed carbonate–silicate terrains [45]. The coexistence of these associations, together with partially lithogenic P and S, suggests that the studied peloids may originate from a mixed carbonate–silicate parent material, consistent with the complex geological structure of spa regions in Serbia, which include sedimentary basins, metamorphic complexes, and granitoid intrusions.

The significant positive correlations among EC and TDS (Figure 2) confirm their common dependence on dissolved ionic species within the sediment–water system. Their strong correlation with Ca, Na, S, and Sr indicates that mineral dissolution and water–rock interaction processes are key controlling mechanisms. The Ca–Sr relationship is particularly indicative of carbonate weathering, since Sr commonly substitutes for Ca in carbonate lattices and is released during dissolution processes [46]. Similarly, the correlation between Na–K and EC/TDS (Figure 2) suggests silicate alteration and ion exchange reactions involving clay minerals, which release alkali metals into pore solutions. These processes are characteristic of hydrothermal and mineral water environments associated with spa systems [47]. The generally weak negative correlations between pH and most metals and radionuclides suggest that slightly acidic-to-neutral conditions enhance element mobility, particularly for divalent cations, by influencing adsorption–desorption equilibria on clay and Fe–Mn oxide surfaces [48]. Thus, pH acts as a secondary modifying factor, while mineralogical composition and water–rock interaction represent primary controls.

Trace elements (Cd, Pb, Zn, Cu, and Ni; Figure 2) exhibited strong intercorrelations, indicating similar geochemical behavior and possible co-association within Fe-Mn oxide and hydroxide phases. Fe–Mn oxides are well known for their high sorption capacity and their role as scavengers of trace metals in sedimentary environments. The correlation of these metals with Fe and Mn suggests adsorption, co-precipitation, or incorporation into secondary oxide phases formed during weathering and redox cycling [49]. In addition to lithogenic sources, localized anthropogenic inputs cannot be excluded, especially for Cd and Pb, but the overall correlation structure indicates that natural geochemical processes dominate their distribution [45]. Hg exhibited weaker correlations with other metals, implying different binding mechanisms, possibly related to organic matter or sulfide phases.

Natural radionuclides (^226^Ra, ^232^Th, ^40^K, ^238^U, and ^235^U) exhibited strong mutual correlations and significant associations with Al, Fe and K. This pattern indicates a predominantly lithogenic origin and confirms their incorporation within mineral fractions rather than representing recent contamination. ^40^K is typically associated with K-bearing silicates such as feldspars and micas, explaining its correlation with K and Al [50]. ^232^Th is commonly immobile and strongly bound to resistant mineral phases, particularly heavy minerals and clays, while ^238^U may occur in accessory minerals or be adsorbed onto Fe oxides. The correlation of ^238^U and ^226^Ra isotopes suggests their derivation from common parent materials, with minor redistribution influenced by differential mobility [51,52]. ^226^Ra, due to its chemical similarity to alkaline earth elements, may substitute for Ca in carbonate minerals, explaining its association with Ca-rich phases. The artificial radionuclide ^137^Cs and unsupported ^210^Pb displayed moderate positive correlations with Fe, Mn, and Zn, suggesting their preferential accumulation in finer sediment fractions and the potential additional influence of atmospheric deposition. Overall, the observed correlation patterns reflect both natural geochemical associations and some additional anthropogenic influences in the studied peloids in Serbia, with Fe–Mn oxide phases and fine-grained materials playing key roles in the accumulation of metals and radionuclides ([45], and references therein).

**Figure 3:**
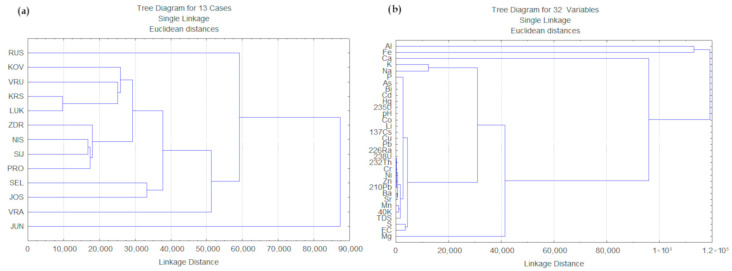
Hierarchical cluster analysis (HCA) dendrograms illustrating similarities among (**a**) sampling sites and (**b**) analyzed elements, radionuclides and physico-chemical parameters based on Euclidean distance.

The hierarchical cluster analysis (HCA) of peloid samples further supported these interpretations (Figure 3a,b). The grouping of samples into three main clusters (Figure 3a) reflects similarities in lithological background, geochemical composition, and physicochemical conditions rather than strict geographical proximity. The clustering of SEL (central Serbia) and JOS (SW Serbia) suggests comparable parent material composition despite spatial separation, whereas the pairing of KOV–VRU (central Serbia, both) and KRS–LUK (SW Serbia) indicates similar carbonate–silicate contributions within central and southwestern Serbia. The second subcluster consists of ZDR and NIS from central Serbia, SIJ from south Serbia, and PRO from SW Serbia. This grouping (Figure 3a) likely reflects mixed lithological influence combined with comparable hydrochemical regimes. The separation of JUN, RUS, and VRA (Figure 3a) suggests distinct geochemical signatures, potentially related to specific hydrothermal inputs, mineralogical differences, or basin morphology influencing sediment accumulation.

Overall, the combined correlation and clustering analyses indicate that the distribution of PTEs, radionuclides, and physicochemical parameters in Serbian peloids (Figure 2 and Figure 3b) is primarily governed by lithogenic factors, including parent-rock composition, carbonate and silicate weathering, clay mineral content, and Fe–Mn oxide phases. Secondary controls include water–rock interaction processes, ion exchange, redox dynamics, and atmospheric deposition. Anthropogenic influence appears limited and site-specific. From a geological perspective, the results reflect the heterogeneous tectono-stratigraphic framework of Serbia, from north to south, where sedimentary basins, metamorphic complexes, and magmatic bodies collectively influence the mineralogical and geochemical characteristics of spa peloids.

### 3.5. Preliminary Peloid Safety Assessment

#### 3.5.1. Toxic and Carcinogenic Elements

**Figure 4:**
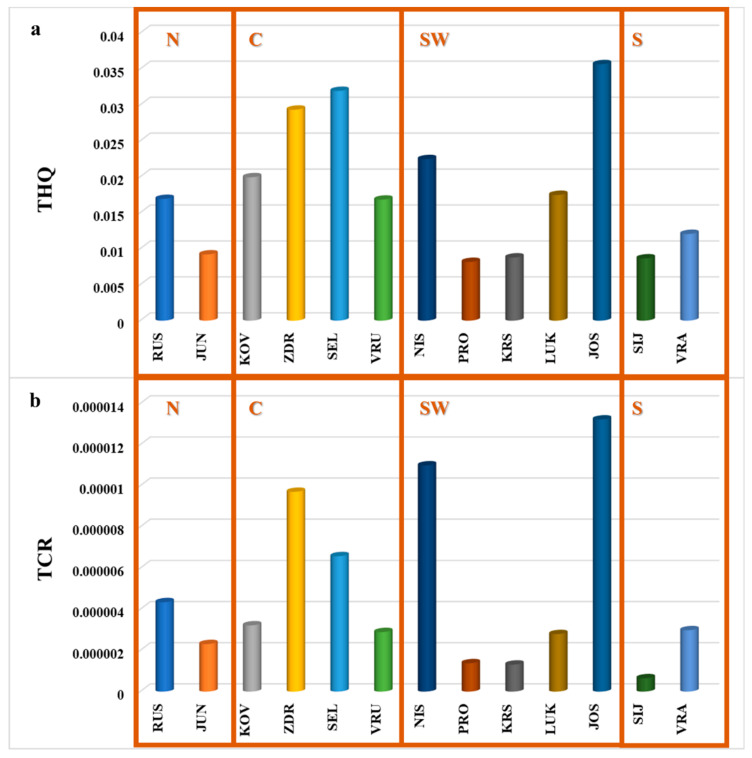
(**a**) The total noncarcinogenic (THQ) and (**b**) the total carcinogenic (TCR) risks for long-term dermal exposure to peloids. Parts of Serbia: N—north; C—central; SW—south-west; S—south.

The human health risk associated with dermal exposure to toxic and carcinogenic elements during therapeutic application of peloids was assessed using integrated risk indicators, as a worst-case scenario investigating the pseudo-total element concentrations. Noncarcinogenic risk was expressed as THQ, while carcinogenic risk was reported as TCR and is presented in Table S2. In this study, these aggregated metrics are presented to provide a concise evaluation of overall dermal risk. For elements that have reference dose (RfD) values and cancer slope factors (CSFs), the total noncarcinogenic and total carcinogenic risks were assessed (Table S2; Figure 4a,b). Based on the determined concentrations, there is no increased noncarcinogenic risk (HI < 1), and the values of carcinogenic risk are acceptable (1 × 10^−6^ < TCR < 1 × 10^−4^) in the worst-case scenario of dermal exposure to peloids. It should be noted that dermal bioavailability is generally low, which may significantly reduce actual exposure compared to the pseudo-total concentrations reported. The HI values are negligible for peloid samples, implying that they are safe for long-term use. Comparing HI between samples, the specific peloids from the central (ZDR) and south-west (NIS and JOS) parts of Serbia have higher HI compared to other investigated peloids. This may be caused by the different lithological influences. The total carcinogenic risk evaluated in the worst-case scenario of exposure implies that there is an acceptable dermal risk for long-term exposure to peloids. The highest THQ was also obtained for the samples ZDR, NIS, and JOS, which were probably most influenced by the geogenic origin of PTEs. According to the available literature, there are no studies investigating dermal risk via long-term exposure to potentially toxic elements from peloids, and this worst-case scenario approach showed that, based on the toxic element concentrations determined in peloids, the investigated samples are safe for use. Still, especially for specific samples, even if the elements originate mostly from a geogenic origin and are less bioaccessible to humans, peloids should be professionally used for medical and spa treatment to provide the greatest benefit to humans and avoid unnecessary exposure to some elements. In a study published by Bastos et al. [53], it was shown that the dermal bioaccessibility of elements from sulfurous peloids was generally very low, indicating minimal systemic dermal uptake and low potential risk for users. On the contrary, in our study, total concentrations were used for risk assessment, and our approach is probably overestimated, but it may be used as a preliminary approach for screening dermal exposure to therapeutic muds/peloids.

#### 3.5.2. Radiation Hazard Parameters

**Figure 5:**
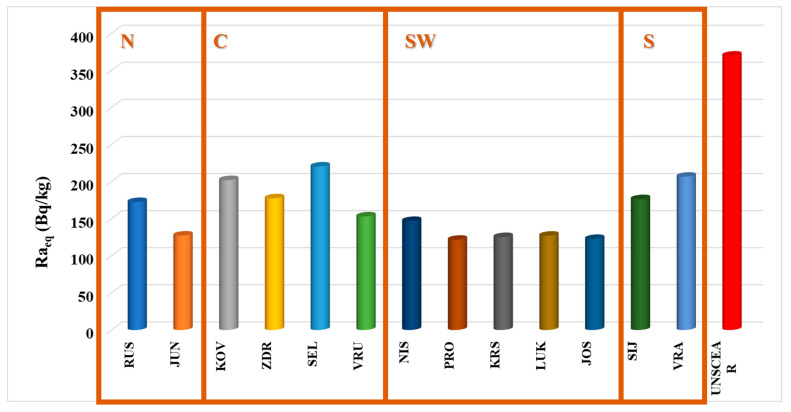
The comparison of radium equivalent activity for peloid samples and UNSCEAR values.

The results of the calculation of health risk assessment parameters for radionuclides are presented in Table 4. It can be observed that values for radium equivalent activity range from 121.63 Bq/kg (PRO) to 220.46 Bq/kg (SEL), and the average value is 161.54 Bq/kg. All calculated values are below the value recommended by UNSCEAR (370 Bq/kg) and are compared in Figure 5 [39].

Another important parameter is the absorbed dose rate (D_out_), which takes into consideration the contribution of gamma radiation from naturally occurring radionuclides ^226^Ra, ^232^Th, and ^40^K, and the calculated values range from 56.33 nGy/h at the Prolom Spa to 94.85 nGy/h at the Vranjska Spa, with an average value of 75.01 nGy/h. The annual effective dose (AED) was calculated, and it can be observed that the average AED value is 92.06 μSv/y, with values ranging from 69.12 μSv/y at the Prolom Spa to 119.61 μSv/y at the Selters Spa. The average value is higher than the annual equivalent world average for outdoor gamma radiation (70 μSv/y), but the value is still below the maximum recommended worldwide (1 mSv/y) [54]. The gamma radiation hazard index (I_ϒ_r), a significant health index that estimates the excess external and indoor gamma radiation, showed that the lowest gamma radiation hazard index value was observed in peloids originating from the Prolom Spa (0.89), and the highest value was observed in peloids from the Selters Spa (1.61), with an average value of 1.2 for all samples. The health hazard risk is as low as possible when the values for H_ex_ and H_in_ are less than unity, and the results are presented in Table 4. As shown, H_ex_ is in the range of 0.33 (Prolom Spa) to 0.60 (Selters Spa), with an average value of 0.44. H_in_ is in the range of 0.41 to 0.78, with an average value of 0.55. The highest ELCR value is observed in the sample from the Selters Spa (0.46), while the lowest value is observed in a few spas (0.27): the Prolom Spa, Kursumlijska Spa, and Josanicka Spa. The average value for ELCR is 0.35. It should be emphasized that the calculated ELCR values represent theoretical lifetime risk estimates based on conservative exposure assumptions and do not imply direct medical confirmation of cancer occurrence. They serve as regulatory screening tools to evaluate whether exposure levels remain within internationally accepted safety limits. All calculated values for the radiological indices (radium equivalent activity, absorbed gamma dose rate, annual effective dose, gamma radiation hazard index, external hazard index, and excess lifetime cancer risk) are lower than the world average or the maximum allowed level defined by UNSCEAR and ICRP.

#### 3.5.3. Therapeutic Relevance and Benefit–Risk Perspective

While the previous sections focus on potential toxicological and radiological risks, it is equally important to consider the well-established therapeutic applications of peloids in order to provide a balanced and scientifically sound assessment. Peloids have long been used in balneotherapy and rehabilitation medicine due to their combined thermal, mechanical, chemical, and, in some cases, radiological properties.

The therapeutic efficacy of peloids is largely attributed to their ability to retain heat, stimulate microcirculation, and promote anti-inflammatory responses [3,55,56]. Macroelements such as Ca and Mg play an important role in skin physiology and cellular metabolism, while sulfur-containing compounds are particularly valued for their keratolytic, antibacterial, and anti-inflammatory effects, making them beneficial in the treatment of dermatological conditions [57,58]. In addition, the physicochemical properties of clay minerals, including cation-exchange capacity, contribute to ion transfer processes at the skin–peloid interface, potentially enhancing therapeutic outcomes [56]. Furthermore, naturally occurring radionuclides in peloids have been discussed in the context of spa therapy, where low-level radiation exposure has been associated with stimulation of biological defense mechanisms, a concept often referred to as radiation hormesis [58]. Although this phenomenon remains a subject of ongoing scientific debate, several studies suggest that low-dose exposure in controlled therapeutic settings may contribute to beneficial effects when combined with other peloid properties [13,56,58].

Therefore, the evaluation of Serbian peloids should not be limited solely to the identification of potentially harmful components but should instead be considered within a broader benefit–risk framework. The results obtained in this study indicate that, despite the presence of certain potentially toxic elements and measurable radioactivity, the overall composition of the investigated peloids remains consistent with materials traditionally used in spa therapy. Consequently, their safe and effective use depends not only on their chemical composition but also on controlled application, exposure time, and adherence to established balneological practices.

## 4. Limitations and Further Perspectives

Although this study provides a comprehensive physicochemical, elemental, and radiological characterization of Serbian spa peloids, several limitations should be noted. The health risk assessment (EDInc, EDIc, THQ, HI, TCR, ELCR, and AED) was based on internationally accepted environmental and radiological models, representing standardized probabilistic screening tools with conservative exposure assumptions. These indicators do not provide direct clinical evidence, and the calculated values reflect theoretical lifetime risks under defined exposure scenarios. Elemental concentrations were determined after aqua regia digestion, representing pseudo-total contents, and actual dermal bioavailability may vary depending on chemical speciation, mineral phases, and exposure conditions. Future studies integrating speciation analysis and mineralogical characterization, and controlled exposure experiments, would provide improved insight into element or radionuclide mobility and bioaccessible fractions, strengthening the framework for evidence-based evaluation of therapeutic-mud safety.

## 5. Conclusions

This study presents a comprehensive physicochemical, elemental, and radiological characterization of peloids from 13 spas across four regions in Serbia, representing the first extensive dataset for the Balkan region. Furthermore, the study combines geochemical characterization with a preliminary human health risk assessment, offering a comprehensive framework for evaluating the safety and applicability of therapeutic muds in regions with limited prior data. The results demonstrate that Serbian peloids exhibit considerable heterogeneity in both macro- and microelement concentrations, reflecting diverse geological formations, hydrochemical conditions, and sediment origins. This variability underscores the importance of site-specific evaluation of peloids when selecting peloids for therapeutic applications.

Elemental analysis indicates that the composition of Serbian peloids is predominantly controlled by geogenic factors, with macro- and trace element distributions consistent with the mineralogical characteristics of the source materials. Concentrations of potentially toxic elements were generally comparable to those reported for therapeutic peloids worldwide, indicating limited anthropogenic influence and highlighting the importance of ongoing monitoring at sites with elevated values. The worst-case scenario assessment of exposure to the potentially toxic and carcinogenic element concentrations indicates no significant noncarcinogenic risks and acceptable carcinogenic risks for adults under long-term, controlled use, supporting the safe dermal application of these peloids under professional supervision.

Gamma spectrometric analysis showed that the activity concentrations of natural radionuclides were within internationally recommended limits, while anthropogenic concentrations of ^137^Cs were negligible. The assessed radiological hazard indices, based on the radionuclide concentrations in peloids, confirmed that the investigated peloids do not pose a significant radiological risk when used under controlled spa or medical use.

Overall, this study establishes a robust chemical and radiological baseline for Serbian peloids, providing one of the most comprehensive datasets available for spa muds in the region. It contributes valuable data to the limited international literature on peloids and supports their safe and evidence-based application in therapeutic and wellness settings. Our findings provide a preliminary framework for future studies on standardization and quality control, and for more detailed investigations focusing on mineralogical characterization, bioaccessibility, and clinical relevance, potentially informing international guidelines for therapeutic peloid use.

## Figures and Tables

**Table 1:** Physicochemical properties (pH, EC, salinity and TDS) of peloid samples.

Sample	pH	EC (μS/cm)	Salinity (%)	TDS (mg/L)
RUS	9.52	6610	4	>1999 (off limit)
JUN	9.13	1278	0.4	742
KOV	8.26	348	<0.1	186
ZDR	7.76	77.5	<0.1	43
SEL	7.92	1140	0.4	763
VRU	6.59	109.8	<0.1	59
NIS	7.08	252	<0.1	147
PRO	7.98	117.9	<0.1	74
KRS	7.15	131.7	<0.1	85
LUK	8.6	218	<0.1	141
JOS	6.68	136.2	<0.1	77
SIJ	9.06	298	<0.1	171
VRA	7.43	302	<0.1	169
Mean	7.94	847.62	1.60	221.42
Max	9.52	6610	4	763
Min	6.59	77.5	0.4	43
Median	7.92	252	0.4	144

**Table 2:** Concentration of macro- and microelements in peloid samples.

Sample	Macroelements (g/kg)								Microelements (mg/kg)														
	Al	Ca	Fe	K	Mg	Na	P	S	As	B	Ba	Bi	Cd	Co	Cr	Cu	Hg	Li	Mn	Ni	Pb	Sr	Zn
RUS	75.69 ± 0.30	72.91 ± 0.75	86.53 ± 0.16	32.32 ± 0.46	47.39 ± 0.35	30.35 ± 0.24	3.035 ± 0.004	8.72 ± 0.03	13.94 ± 0.06	118.77 ± 0.65	124.10 ± 2.52	1.96 ± 0.18	0.82 ± 0.02	14.03 ± 0.05	51.93 ± 0.61	42.39 ± 0.48	0.013 ± 0.001	27.30 ± 0.16	689.9 ± 7.8	47.18 ± 0.08	24.22 ± 0.25	98.3 ± 2.4	118.37 ± 0.31
JUN	8.95 ± 0.25	100.66 ± 0.50	16.49 ± 0.31	1.65 ± 0.63	17.58 ± 0.04	2.62 ± 0.14	0.639 ± 0.004	2.71 ± 0.02	7.73 ± 0.35	10.70 ± 0.14	55.24 ± 0.33	17.25 ± 1.68	1.55 ± 0.03	6.12 ± 0.02	18.02 ± 0.26	14.64 ± 0.17	0.23 ± 0.03	13.98 ± 0.07	391.3 ± 3.5	15.50 ± 0.05	8.8 ± 1.1	152.10 ± 0.38	34.88 ± 0.14
KOV	58.05 ± 0.76	41.72 ± 0.16	89.6 ± 1.3	7.78 ± 0.07	20.15 ± 0.29	2.58 ± 0.04	0.672 ± 0.002	2.67 ± 0.01	8.42 ± 0.21	<0.06	96.4 ± 1.8	2.39 ± 0.57	0.73 ± 0.01	19.70 ± 0.08	95.6 ± 1.3	28.81 ± 0.51	0.0160 ± 0.0007	30.60 ± 0.15	653 ± 12	117.70 ± 0.21	19.67 ± 0.13	47.44 ± 1.01	90.14 ± 0.16
ZDR	27.37 ± 0.29	6.96 ± 0.05	28.31 ± 0.21	2.34 ± 0.48	4.94 ± 0.09	0.87 ± 0.10	0.319 ± 0.004	0.296 ± 0.001	33.49 ± 0.43	3.03 ± 0.01	117.84 ± 0.76	35.20 ± 2.16	3.52 ± 0.01	15.77 ± 0.04	44.34 ± 0.34	91.12 ± 0.55	0.53 ± 0.03	23.88 ± 0.25	969.5 ± 5.9	32.01 ± 0.09	59.73 ± 0.30	28.50 ± 0.19	103.57 ± 0.18
SEL	64.59 ± 0.03	8.77 ± 0.04	100.90 ± 0.53	4.65 ± 0.02	9.76 ± 0.06	9.50 ± 0.06	0.48 ± 0.01	0.793 ± 0.001	19.31 ± 0.24	<0.06	85.09 ± 0.71	2.94 ± 0.73	0.9772 ± 0.0004	24.07 ± 0.07	132.67 ± 0.49	35.65 ± 0.21	0.022 ± 0.002	34.50 ± 0.54	1152 ± 12	148.33 ± 0.61	49.11 ± 0.22	58.61 ± 0.71	119.93 ± 0.51
VRU	43.94 ± 0.10	8.10 ± 0.03	51.13 ± 0.18	4.65 ± 0.01	12.46 ± 0.18	1.708 ± 0.003	0.69 ± 0.02	0.51 ± 0.01	7.87 ± 0.17	<0.06	92.39 ± 0.86	0.84 ± 9.68	0.63 ± 0.01	15.24 ± 0.14	76.93 ± 0.91	18.18 ± 0.25	0.017 ± 0.002	27.42 ± 0.08	591.5 ± 6.1	98.52 ± 0.92	13.33 ± 0.09	23.26 ± 0.14	60.30 ± 0.47
NIS	40.01 ± 0.36	20.02 ± 0.21	11.11 ± 0.08	7.45 ± 0.34	7.37 ± 0.14	4.07 ± 0.12	0.140 ± 0.002	0.554 ± 0.005	39.32 ± 0.23	15.01 ± 0.09	717.46 ± 7.49	16.37 ± 4.82	1.14 ± 0.01	1.93 ± 0.04	8.90 ± 0.35	18.59 ± 0.12	0.21 ± 0.05	6.04 ± 0.04	257.2 ± 2.8	7.41 ± 0.03	27.54 ± 0.16	513.4 ± 6.8	43.59 ± 0.20
PRO	18.87 ± 0.11	13.16 ± 0.08	13.26 ± 0.11	1.04 ± 0.91	10.22 ± 0.06	1.14 ± 0.14	0.857 ± 0.004	0.364 ± 0.004	4.21 ± 0.34	0.84 ± 0.02	73.35 ± 1.32	15.59 ± 1.31	1.27 ± 0.01	7.8 ± 0.06	22.91 ± 0.41	15.61 ± 0.14	0.29 ± 0.09	7.70 ± 0.05	511.6 ± 4.5	9.36 ± 0.02	11.38 ± 0.36	44.49 ± 0.34	30.72 ± 0.09
KRS	48.58 ± 0.13	31.30 ± 0.08	69.14 ± 0.04	2.47 ± 0.02	24.86 ± 0.26	2.340 ± 0.002	1.439 ± 0.004	0.215 ± 0.004	3.54 ± 0.46	<0.06	54.93 ± 0.97	1.52 ± 0.38	0.50 ± 0.01	9.41 ± 0.09	35.68 ± 0.61	13.42 ± 0.23	0.011 ± 0.002	6.86 ± 0.11	556.5 ± 7.2	19.63 ± 0.16	11.59 ± 0.24	30.75 ± 0.61	41.28 ± 0.31
LUK	44.15 ± 0.15	24.64 ± 0.12	68.36 ± 0.66	4.81 ± 0.05	20.07 ± 0.19	2.90 ± 0.02	1.668 ± 0.004	0.233 ± 0.007	7.12 ± 0.06	<0.06	70.26 ± 0.12	0.80 ± 0.72	0.628 ± 0.003	10.56 ± 0.07	88.69 ± 0.42	19.31 ± 0.18	0.010 ± 0.001	11.07 ± 0.08	513.0 ± 6.4	45.64 ± 0.26	25.18 ± 0.24	33.25 ± 0.43	66.13 ± 0.35
JOS	44.63 ± 0.15	9.01 ± 0.02	124.55 ± 0.39	6.77 ± 0.08	19.45 ± 0.19	2.578 ± 0.001	1.357 ± 0.008	0.70 ± 0.02	45.26 ± 0.64	<0.06	59.23 ± 0.94	3.08 ± 0.18	0.89 ± 0.01	27.28 ± 0.16	72.2 ± 1.1	18.21 ± 0.24	0.018 ± 0.002	9.69 ± 0.13	960 ± 14	75.32 ± 0.39	17.06 ± 0.23	23.19 ± 0.34	74.31 ± 0.44
SIJ	28.93 ± 0.22	16.29 ± 0.15	19.51 ± 0.09	5.47 ± 0.59	8.06 ± 0.11	12.55 ± 0.08	0.62 ± 0.03	0.44 ± 0.03	1.23 ± 0.90	22.04 ± 0.21	633.57 ± 4.62	22.52 ± 4.93	1.92 ± 0.01	8.68 ± 0.05	33.48 ± 0.34	27.97 ± 0.21	2.35 ± 0.62	27.24 ± 0.18	362.9 ± 2.4	22.82 ± 0.05	13.49 ± 0.21	289.1 ± 1.7	60.09 ± 0.04
VRA	78.19 ± 0.39	41.83 ± 0.22	34.64 ± 0.08	14.58 ± 0.08	14.51 ± 0.08	16.50 ± 0.10	0.793 ± 0.007	0.43 ± 0.01	9.12 ± 0.22	<0.06	762.7 ± 3.5	<0.54	0.26 ± 0.01	5.45 ± 0.07	49.21 ± 0.34	14.52 ± 0.02	0.018 ± 0.002	11.40 ± 0.04	213.5 ± 1.4	30.01 ± 0.35	10.51 ± 0.19	459.3 ± 6.7	42.53 ± 0.54
Mean	44.77	30.41	54.89	7.38	16.68	6.90	0.98	1.43	15.43	28.40	226.35	10.04	1.14	12.77	56.20	27.57	0.29	18.28	601.68	51.49	22.43	138.59	68.14
Standard deviation	0.25	0.19	0.32	0.29	0.16	0.08	0.01	0.01	0.33	0.19	1.99	2.28	0.01	0.07	0.58	0.25	0.06	0.15	6.61	0.25	0.29	1.67	0.29
Max	78.19	100.66	124.55	32.32	47.39	30.35	3.035	8.72	45.26	118.77	762.7	35.2	3.52	27.28	132.67	91.12	2.35	34.5	1152	148.33	59.73	513.4	119.93
Min	8.95	6.96	11.11	1.04	4.94	0.87	0.14	0.215	1.23	0.84	54.93	0.8	0.26	1.93	8.9	13.42	0.01	6.04	213.5	7.41	8.8	23.19	30.72
Median	44.15	20.02	51.13	4.81	14.51	2.62	0.69	0.51	8.42	12.86	92.39	3.01	0.89	10.56	49.21	18.59	0.018	13.98	556.5	32.01	17.06	47.44	60.30

**Table 3:** Radionuclide concentrations in peloid samples.

Sample	Radionuclide Concentrations (Bq/kg)						
	^226^Ra	^232^Th	^40^K	^238^U	^235^U	^137^Cs	^210^Pb
RUS	28 ± 1	44 ± 3	1060 ± 70	44 ± 5	2.8 ± 0.3	47 ± 3	160 ± 10
JUN	37 ± 2	40 ± 3	430 ± 30	38 ± 4	2.3 ± 0.2	<0.06	38 ± 4
KOV	68 ± 4	55 ± 5	720 ± 50	46 ± 10	4.0 ± 0.4	<0.3	53 ± 9
ZDR	42 ± 2	63 ± 4	590 ± 40	44 ± 5	3.1 ± 0.3	1.4 ± 0.3	44 ± 5
SEL	68 ± 3	70 ± 5	680 ± 40	76 ± 7	5.8 ± 0.4	<0.05	74 ± 7
VRU	42 ± 2	47 ± 5	570 ± 40	43 ± 9	2.3 ± 0.3	<0.3	47 ± 8
NIS	26 ± 1	62 ± 5	420 ± 30	36 ± 6	1.9 ± 0.2	<0.09	36 ± 5
PRO	30 ± 2	42 ± 4	410 ± 30	37 ± 8	2.3 ± 0.3	<0.3	27 ± 6
KRS	30 ± 1	44 ± 3	420 ± 30	38 ± 4	2.6 ± 0.2	<0.05	27 ± 4
LUK	30 ± 2	41 ± 4	500 ± 40	53 ± 8	2.7 ± 0.3	1.6 ± 0.4	32 ± 6
JOS	32 ± 1	36 ± 3	510 ± 30	29 ± 4	1.3 ± 0.1	7.0 ± 0.6	33 ± 4
SIJ	31 ± 2	64 ± 4	700 ± 40	30 ± 5	1.6 ± 0.2	<0.05	22 ± 4
VRA	54 ± 2	75 ± 5	590 ± 40	23 ± 4	1.0 ± 0.1	<0.06	22 ± 4
Mean	40	53	590	41	2.6	14.3	47
Standard Deviation	2	4	40	6	0.3	1.1	6
Max	68	75	1060	76	5.8	47	160
Min	26	36	410	23	1	1.4	22
Median	32	47	570	38	2.3	4.3	36

**Table 4:** Radiation hazard parameters: radium equivalent activity, absorbed dose rate, annual effective dose, gamma index, external hazard index, and excess lifetime risk of cancer for peloid samples.

Sample	Ra_eq_ (Bq/kg)	D_out_ (nGy/h)	AED (μSv/y)	I_ϒr_	H_ex_	H_in_	ELCR (×10^−3^)
RUS	172.54	83.71	102.74	1.33	0.47	0.54	0.40
JUN	127.31	59.19	72.63	0.93	0.34	0.44	0.28
KOV	202.09	94.66	116.17	1.48	0.55	0.73	0.45
ZDR	177.52	82.06	100.71	1.30	0.51	0.62	0.39
SEL	220.46	97.47	119.61	1.61	0.60	0.78	0.46
VRU	153.1	71.56	87.82	1.13	0.41	0.53	0.34
NIS	147	66.97	82.19	1.07	0.40	0.47	0.32
PRO	121.63	56.33	69.12	0.89	0.33	0.41	0.27
KRS	125.26	57.95	71.12	0.92	0.34	0.42	0.27
LUK	127.13	59.47	72.99	0.94	0.34	0.42	0.28
JOS	122.75	57.80	70.93	0.91	0.33	0.42	0.27
SIJ	176.42	82.17	100.84	1.31	0.48	0.56	0.39
VRA	206.68	94.85	116.40	1.50	0.56	0.70	0.45
Mean	159.99	74.17	91.02	1.18	0.43	0.54	0.35

## Data Availability

The original contributions presented in this study are included in the article. Further inquiries can be directed to the corresponding author.

## Supplementary Materials

The following supporting information can be downloaded at: https://www.mdpi.com/article/10.3390/toxics14050355/s1, Section S1. Study area; Table S1. Activity ratio; Table S2. The total noncarcinogenic (THQ) and total carcinogenic (TCR) risks for long-term dermal exposure to peloids. References [59,60,61,62,63,64] are citied in the Supplementary Materials.

## References

[1] Carretero I. (2002). Clay Minerals and Their Beneficial Effects upon Human Health. A Review. Appl. Clay Sci..

[2] Knorst-Fouran A., Casás L.M., Legido J.L., Coussine C., Bessières D., Plantier F., Lagière J., Dubourg K. (2012). Influence of Dilution on the Thermophysical Properties of Dax Peloid (TERDAX^®^). Thermochim. Acta.

[3] Maraver F., Armijo F., Fernandez-Toran M.A., Armijo O., Ejeda J.M., Vazquez I., Corvillo I., Torres-Piles S. (2021). Peloids as Thermotherapeutic Agents. Int. J. Environ. Res. Public Health.

[4] Tateo F., Summa V. (2007). Element Mobility in Clays for Healing Use. Appl. Clay Sci..

[5] Fioravanti A., Cantarini L., Guidelli G.M., Galeazzi M. (2011). Mechanisms of Action of Spa Therapies in Rheumatic Diseases: What Scientific Evidence Is There?. Rheumatol. Int..

[6] Gomes C.d.S.F. (2018). Healing and Edible Clays: A Review of Basic Concepts, Benefits and Risks. Environ. Geochem. Health.

[7] Cozzi F., Ciprian L., Carrara M., Galozzi P., Zanatta E., Scanu A., Sfriso P., Punzi L. (2018). Balneotherapy in Chronic Inflammatory Rheumatic Diseases—A Narrative Review. Int. J. Biometeorol..

[8] Calin M.R., Radulescu I., Ion A.C., Capra L., Almasan E.R. (2020). Investigations on Chemical Composition and Natural Radioactivity Levels from Salt Water and Peloid Used in Pelotherapy from the Techirghiol Lake, Romania. Environ. Geochem. Health.

[9] Park C., Kim J.-H., Choi W., Kim D., No S.-G., Chung D., Lee H., Seo S., Seo S.M. (2024). Natural Peloids Originating from Subsea Depths of 200 m in the Hupo Basin, South Korea: Physicochemical Properties for Potential Pelotherapy Applications. Environ. Geochem. Health.

[10] da Silva P.S.C., Torrecilha J.K., de Macedo Gouvea P.F., Máduar M.F., de Oliveira S.M.B., Scapin M.A. (2015). Chemical and Radiological Characterization of Peruíbe Black Mud. Appl. Clay Sci..

[11] Veniale F., Barberis E., Carcangiu G., Morandi N., Setti M., Tamanini M., Tessier D. (2004). Formulation of Muds for Pelotherapy: Effects of “Maturation” by Different Mineral Waters. Appl. Clay Sci..

[12] Torrecilha J.K., Mendes A.P.T., Theophilo C.Y.S., da Silva Matias Dantas Linhares H.M., de Paula J.H., Scapin M.A., Garcia R.H.L., Maraver F., da Silva P.S.C. (2023). Characterization of Peloids from Different Regions of Brazil. J. Trace Elem. Miner..

[13] Roca Jalil M.E., Sanchez M., Pozo M., Soria C.O., Vela L., Gurnik N., Baschini M. (2020). Assessment of Natural and Enhanced Peloids from the Copahue Thermal System (Argentina): Effects of the Drying Procedure on Lidocaine Adsorption. Appl. Clay Sci..

[14] Carretero M.I., Pozo M., Legido J.L., Fernández-González M.V., Delgado R., Gómez I., Armijo F., Maraver F. (2014). Assessment of Three Spanish Clays for Their Use in Pelotherapy. Appl. Clay Sci..

[15] Pozo M., Carretero M.I., Maraver F., Pozo E., Gómez I., Armijo F., Rubí J.A.M. (2013). Composition and Physico-Chemical Properties of Peloids Used in Spanish Spas: A Comparative Study. Appl. Clay Sci..

[16] Batnasan B., Gankhurel G., Gania D. (2021). Chemical Composition of Peloid from Lake Khyargas. Proceedings of the 5th International Conference on Chemical Investigation and Utilization of Natural Resource (ICCIUNR-2021).

[17] Özay P., Karagülle M., Kardeş S., Karagülle M.Z. (2020). Chemical and Mineralogical Characteristics of Peloids in Turkey. Environ. Monit. Assess..

[18] Alzahrani A.J., Redwan O.K., Chieb M., El-Saeid M.H. (2025). Water and Soil Physico-Chemical Characteristics in Ibex Reserve: An Environmental Case Study of Houta Bani Tamim. Sustainability.

[19] (2017). General Requirements for the Competence of Testing and Calibration Laboratories.

[20] Vukanac I., Đurašević M., Nikolić J.K., Pantelić G., Rajačić M., Janković M., Sarap N., Todorović D. (2021). Preparation and Validation of Laboratory Radioactive Standards for Experimental Calibration in Gamma Ray Spectrometry. Radiat. Phys. Chem..

[21] RAIS Risk Assessment Information System. https://Rais.Ornl.Gov/.

[22] Milićević T., Urošević M.A., Relić D., Vuković G., Škrivanj S., Popović A. (2018). Bioavailability of Potentially Toxic Elements in Soil–Grapevine (Leaf, Skin, Pulp and Seed) System and Environmental and Health Risk Assessment. Sci. Total Environ..

[23] Mitić J., Relić D., Pucarević M., Stojić N., Štrbac S., Ninkov J., Milićević T. (2025). The Oral Bioaccessibility of Potentially Toxic Elements of Illegal Landfills’ Soil and Health Risk Assessment for Field Workers. Chemosphere.

[24] Adewoyin O.O., Omeje M., Omonhinmin C., Nwinyi O., Arijaje T., Ayanbisi O. (2023). Assessment of Radium Equivalent Activity and Total Annual Effective Dose in Cassava Cultivated around Ewekoro Cement Factory. J. Food Prot..

[25] Caridi F., Di Bella M., Sabatino G., Belmusto G., Fede M.R., Romano D., Italiano F., Mottese A.F. (2021). Assessment of Natural Radioactivity and Radiological Risks in River Sediments from Calabria (Southern Italy). Appl. Sci..

[26] Adewoyin O.O., Omeje M., Joel E.S., Akinwumi S.A., Ehi-Eromoseled C.O., Embong Z. (2018). Radionuclides Proportion and Radiological Risk Assessment of Soil Samples Collected in Covenant University, Ota, Ogun State Nigeria. MethodsX.

[27] Eke B.C., Akomolafe I.R., Ukewuihe U.M., Onyenegecha C.P. (2024). Assessment of Radiation Hazard Indices Due to Natural Radionuclides in Soil Samples from Imo State University, Owerri, Nigeria. Environ. Health Insights.

[28] Mbonu C.C., Essiett A.A., Ben U.C. (2021). Geospatial assessment of radiation hazard indices soil samples from Njaba, Imo State, South-Eastern Nigeria. Environ. Chall..

[29] Craw D., Rufaut C., Pillai D. (2022). Geological Controls on Evolution of Evaporative Precipitates on Soil-Free Substrates and Ecosystems, Southern New Zealand. Sci. Total Environ..

[30] Jurecek L., Rajcigelova T., Kozarova A., Werner T., Vormann J., Kolisek M. (2021). Beneficial Effects of an Alkaline Topical Treatment in Patients with Mild Atopic Dermatitis. J. Cosmet. Dermatol..

[31] Lukić M., Pantelić I., Savić S.D. (2021). Towards Optimal PH of the Skin and Topical Formulations: From the Current State of the Art to Tailored Products. Cosmetics.

[32] Kaufhold S., Dohrmann R., Klinkenberg M., Noell U. (2015). Electrical Conductivity of Bentonites. Appl. Clay Sci..

[33] Carbajo J.M., Maraver F. (2018). Salt Water and Skin Interactions: New Lines of Evidence. Int. J. Biometeorol..

[34] Bastos C.M., Rocha F. (2023). Experimental Peloid Formulation Using a Portuguese Bentonite and Different Mineral-Medicinal Waters Suitable for Therapeutic and Well-Being Purposes. Clays Clay Miner..

[35] Shaltout A.A., Ahmed S.I., Abayazeed S.D., El-Taher A., Abd-Elkader O.H. (2017). Quantitative Elemental Analysis and Natural Radioactivity Levels of Mud and Salt Collected from the Dead Sea, Jordan. Microchem. J..

[36] Lampropoulou P., Petrounias P., Rogkala A., Giannakopoulou P.P., Gianni E., Mantzoukas S., Lagogiannis I., Koukouzas N., Hatziantoniou S., Papoulis D. (2023). Microstructural and Microbiological Properties of Peloids and Clay Materials from Lixouri (Kefalonia Island, Greece) Used in Pelotherapy. Appl. Sci..

[37] Mrvić V.V., Saljnikov E., Sikirić B., Jaramaz D. (2022). Concentration, Background Values and Limits of Potential Toxic Elements in Soils of Central Serbia. Advances in Understanding Soil Degradation.

[38] Ullrich S.M., Tanton T.W., Abdrashitova S.A. (2001). Mercury in the Aquatic Environment: A Review of Factors Affecting Methylation. Crit. Rev. Environ. Sci. Technol..

[39] United Nations Scientific Committee on the Effects of Atomic Radiation (2000). Sources and Effects of Ionizing Radiation, United Nations Scientific Committee on the Effects of Atomic Radiation (UNSCEAR) 2000 Report.

[40] Karakaya M.Ç., Doğru M., Karakaya N., Vural H.C., Kuluöztürk F., Bal S.Ş. (2015). Radioactivity Concentrations and Dose Assessments of Therapeutic Peloids from Some Turkish Spas. Clay Miner..

[41] Montaña M., Camacho A., Devesa R., Vallés I., Céspedes R., Serrano I., Blàzquez S., Barjola V. (2013). The Presence of Radionuclides in Wastewater Treatment Plants in Spain and Their Effect on Human Health. J. Clean. Prod..

[42] Vukašinović I., Todorović D., Životić L., Kaluđerović L., Đorđević A. (2018). An Analysis of Naturally Occurring Radionuclides and 137Cs in the Soils of Urban Areas Using Gamma-Ray Spectrometry. Int. J. Environ. Sci. Technol..

[43] Navas A., Gaspar L., López-Vicente M., Machín J. (2011). Spatial Distribution of Natural and Artificial Radionuclides at the Catchment Scale (South Central Pyrenees). Radiat. Meas..

[44] Kuzmanović P., Forkapić S., Mrđa D., Hansman J., Radić J.K. (2025). The Impact of Depleted Uranium on the Environment in Serbia. Sci. Total Environ..

[45] Kabata-Pendias A., Mukherjee A.B. (2007). Trace Elements from Soil to Human.

[46] Chang C., Beckford H.O., Ji H. (2022). Indication of Sr Isotopes on Weathering Process of Carbonate Rocks in Karst Area of Southwest China. Sustainability.

[47] Fulignati P. (2020). Clay Minerals in Hydrothermal Systems. Minerals.

[48] Orucoglu E., Grangeon S., Gloter A., Robinet J.-C., Madé B., Tournassat C. (2022). Competitive Adsorption Processes at Clay Mineral Surfaces: A Coupled Experimental and Modeling Approach. ACS Earth Space Chem..

[49] Zhang C., Yu Z., Zeng G., Jiang M., Yang Z., Cui F., Zhu M., Shen L., Hu L. (2014). Effects of Sediment Geochemical Properties on Heavy Metal Bioavailability. Environ. Int..

[50] Wang K., Li W., Li S., Tian Z., Koefoed P., Zheng X.-Y. (2021). Geochemistry and Cosmochemistry of Potassium Stable Isotopes. Geochemistry.

[51] Molla S., Kumar R., Singhal P., Srivastava V.S., Jha S.K. (2024). Physicochemical and Spatial Distribution Patterns of Radon and Its Parent Radionuclides in the Uranium-Mineralized Singhbhum Region, India. J. Radioanal. Nucl. Chem..

[52] Cumberland S.A., Douglas G., Grice K., Moreau J.W. (2016). Uranium Mobility in Organic Matter-Rich Sediments: A Review of Geological and Geochemical Processes. Earth-Sci. Rev..

[53] Bastos C.M., Rocha F., Patinha C., Marinho-Reis P. (2023). Bioaccessibility by Perspiration Uptake of Minerals from Two Different Sulfurous Peloids. Environ. Geochem. Health.

[54] Huang W.-H., Chen Z.-M., Chen T.-C., Yeh Y.-L. (2025). Assessing Radiological Risks of Natural Radionuclides on Sustainable Campus Environment. Sustainability.

[55] Mefteh S., Lazaar K., Pullar R.C., Medhioub M., Rocha F. (2025). Influence of Solid–Liquid Compositions and Contact Time during Maturation on the Properties of Artificial Peloids for Use in Pelotherapy. Clay Miner..

[56] Carretero M.I. (2020). Clays in Pelotherapy. A Review. Part I: Mineralogy, Chemistry, Physical and Physicochemical Properties. Appl. Clay Sci..

[57] Bastos C.M., Rocha F., Patinha C., Marinho-Reis P. (2024). Characterization of Percutaneous Absorption of Calcium, Magnesium, and Potentially Toxic Elements in Two Tailored Sulfurous Therapeutic Peloids: A Comprehensive in Vitro Pilot Study. Int. J. Biometeorol..

[58] Mourelle M.L., Gómez C.P., Legido J.L. (2024). Peloids in Skin Care and Cosmeceuticals. Cosmetics.

[59] Schmid S.M., Bernoulli D., Fügenschuh B., Matenco L., Schefer S., Schuster R., Tischler M., Ustaszewski K. (2008). The Alpine-Carpathian-Dinaridic Orogenic System: Correlation and Evolution of Tectonic Units. Swiss J. Geosci..

[60] Petrović T., Zlokolica-Mandić M., Veljković N., Vidojević D. (2010). Hydrogeological Conditions for the Forming and Quality of Mineral Waters in Serbia. J. Geochem. Explor..

[61] Vidaković D., Krizmanić J., Dojčinović B.P., Pantelić A., Gavrilović B., Živanović M., Novaković B., Ćirić M. (2019). Alkaline Soda Lake Velika Rusanda (Serbia): The First Insight into Diatom Diversity of This Extreme Saline Lake. Extremophiles.

[62] Marinkovic G., Papic P., Dragisic V., Andrijasevic J. (2016). Hydrogeologic Structures in Two Serbian Spa Towns—Sijarinska Banja and Selters Banja. Geol. Anal. Balk. Poluostrva.

[63] Vukićević E., Burazer N., Roganović J., Mutić T., Veselinović G., Jovančićević B., Gajica G. (2026). Biomarkers for Tracking Organic Matter Maturity in Therapeutic Muds (Peloids): A Comparison of Natural and Spa-Scaled Systems. Water.

[64] Poznanović Spahić M., Marinković G., Spahić D., Sakan S., Jovanić I., Magazinović M., Obradović N. (2023). Water–Rock Interactions across Volcanic Aquifers of the Lece Andesite Complex (Southern Serbia): Geochemistry and Environmental Impact. Water.

