# Supplementary material for: Chemical and Radiological Characterization of Serbian Peloids: Implications for Therapeutic Safety
Source: Toxics. 2026 Apr 23;14(5):355. doi: 10.3390/toxics14050355 (PMC13211522; doi:10.3390/toxics14050355)
Supplement: Supplementary file 1 [file toxics-14-00355-s001.zip › toxics-4245512-supplementary.pdf]

Supplementary Material

Chemical and Radiological Characterization of Serbian Peloids: Implications for Therapeutic Safety

Tijana Mutić [1,*], Tijana Milićević [2], Emilija Vukićević [3], Jovana Roganović [3], Gorica Veselinović [1], Marija Janković [4] and Gordana Gajica [1]

[1] Institute of Chemistry, Technology and Metallurgy—National Institute of the Republic of Serbia, University of Belgrade, Njegoševa 12, 11000 Belgrade, Serbia; gorica.veselinovic@ihtm.bg.ac.rs (G.V.); gordana.gajica@ihtm.bg.ac.rs (G.G.)
[2] Institute of Physics Belgrade—National Institute of the Republic of Serbia, University of Belgrade, Pregrevica 118, 11080 Belgrade, Serbia; tijana.milicevic@ipb.ac.rs
[3] Faculty of Chemistry, University of Belgrade, Studentski trg 12–16, 11158 Belgrade, Serbia; emilija@chem.bg.ac.rs (E.V.); jovanaorlic@chem.bg.ac.rs (J.R.)
[4] VINČA Institute of Nuclear Sciences—National Institute of the Republic of Serbia, University of Belgrade, Mike Petrovića Alasa 12–14, 11000 Belgrade, Serbia; marijam@vin.bg.ac.rs
* Correspondence: tijana.mutic@ihtm.bg.ac.rs

Section S1. Study area

Serbia is characterized by pronounced geological, geomorphological, and hydrogeological heterogeneity, resulting from the interaction of Alpine, Dinaric, and Carpathian-Balkan tectonic units [1]. This geological complexity has led to the formation of diverse mineral and thermal water systems, as well as clay- and sediment-rich environments suitable for peloid formation. The north region, represented by Junaković (JUN) and Rusanda (RUS) spas, is situated within the Pannonian Basin, which is dominated by Neogene and Quaternary sedimentary deposits rich in clays, carbonates, and evaporitic minerals [2]. Rusanda Spa is associated with Lake Rusanda, a shallow saline lake characterized by high salinity (approximately 40-60%), providing a unique geochemical environment for peloid formation [3].

Central Serbia includes Koviljača (KOV), Selters (SEL), Vrujci (VRU), and Ždrelo (ZDR) spas. This region exhibits complex geological conditions, consisting mainly of sedimentary formations intercalated with magmatic and metamorphic rocks. Mineral waters in this area are typically bicarbonate-rich and influenced by both shallow and deep hydrogeological circulation systems [4]. The interaction between mineral waters and clay-rich sediments contributes to the formation and maturation of peloids, which are used in therapeutic applications [5].

The southwestern region, encompassing Jošanička (JOS), Lukovska (LUK), Niška (NIS), Kuršumlijska (KRS), and Prolom (PRO) spas, is strongly influenced by Neogene volcanic activity and associated hydrothermal processes. Geological formations in this area include altered volcanic rocks, mineralized zones, and hydrothermally altered soils, which play a crucial role in controlling the elemental and

radionuclide composition of peloids. Mineral waters from these spas are often enriched in sulfur species, alkali elements, and trace metals, reflecting intensive water–rock interaction [6].

Southern Serbia is represented by Sijarinska (SIJ) and Vranjska (VRA) spas, located within tectonically active zones associated with deep geothermal systems. These areas are characterized by high-temperature mineral waters, elevated gas contents, and prolonged hydrothermal circulation, which significantly influence the geochemical characteristics of the peloid materials [6].

Depending on local spa practice, peloids are obtained either through the mixing of natural geological material (predominantly clay- and zeolite-rich soils) with mineral water or through natural maturation in mud pools and aquatic environments. All analyzed peloid samples are currently used in balneotherapy and medical treatments, ensuring that the investigated materials are representative of those applied in real therapeutic practice.

Table S1. Activity ratio

Sample	$^{238}U/^{226}Ra$	$^{232}Th/^{226}Ra$
RUS	1.57	1.57
JUN	1.03	1.08
KOV	0.68	0.81
ZDR	1.05	1.50
SEL	1.12	1.03
VRU	1.02	1.12
NIS	1.38	2.38
PRO	1.23	1.40
KRS	1.27	1.47
LUK	1.77	1.37
JOS	0.91	1.13
SIJ	0.97	2.06
VRA	0.43	1.39
Mean	1.11	1.41

Table S2. The total noncarcinogenic (THQ) and total carcinogenic (TCR) risks for long-term dermal exposure to peloids

Region	Sample	THQ	TCR
North (N)	RUS	0.0169	4.35E-06
	JUN	0.0092	2.31E-06
Central (C)	KOV	0.0199	3.22E-06
	ZDR	0.0293	9.71E-06
	SEL	0.032	6.59E-06
	VRU	0.0168	2.90E-06
South-West(SW)	NIS	0.0225	1.10E-05
	PRO	0.0082	1.38E-06
	KRS	0.0088	1.31E-06

	LUK	0.0175	2.80E-06
	JOS	0.0357	1.32E-05
South (S)	SIJ	0.0087	6.51E-07
	VRA	0.0121	2.99E-06

References

1. Schmid, S.M.; Bernoulli, D.; Fügenschuh, B.; Matenco, L.; Schefer, S.; Schuster, R.; Tischler, M.; Ustaszewski, K. The Alpine-Carpathian-Dinaridic Orogenic System: Correlation and Evolution of Tectonic Units. *Swiss J. Geosci.* **2008**, *101*, 139–183, doi:10.1007/s00015-008-1247-3.

2. Petrović, T.; Zlokolica-Mandić, M.; Veljković, N.; Vidojević, D. Hydrogeological Conditions for the Forming and Quality of Mineral Waters in Serbia. *J. Geochem. Explor.* **2010**, *107*, 373–381, doi:10.1016/j.gexplo.2010.07.009.

3. Vidaković, D.; Krizmanić, J.; Dojčinović, B.P.; Pantelić, A.; Gavrilović, B.; Živanović, M.; Novaković, B.; Ćirić, M. Alkaline Soda Lake Velika Rusanda (Serbia): The First Insight into Diatom Diversity of This Extreme Saline Lake. *Extremophiles* **2019**, doi:10.1007/s00792-019-01088-6.

4. Marinkovic, G.; Papic, P.; Dragisic, V.; Andrijasevic, J. Hydrogeologic Structures in Two Serbian Spa Towns - Sijarinska Banja and Selters Banja. *Geoloski anali Balkanskoga poluostrva* **2016**, 23–32, doi:10.2298/GABP1677023M.

5. Vukićević, E.; Burazer, N.; Roganović, J.; Mutić, T.; Veselinović, G.; Jovančićević, B.; Gajica, G. Biomarkers for Tracking Organic Matter Maturity in Therapeutic Muds (Peloids): A Comparison of Natural and Spa-Scaled Systems. *Water (Basel).* **2026**, *18*, 457, doi:10.3390/w18040457.

6. Poznanović Spahić, M.; Marinković, G.; Spahić, D.; Sakan, S.; Jovanić, I.; Magazinović, M.; Obradović, N. Water–Rock Interactions across Volcanic Aquifers of the Lece Andesite Complex (Southern Serbia): Geochemistry and Environmental Impact. *Water (Basel).* **2023**, *15*, 3653, doi:10.3390/w15203653.
